# Electroconvulsive Therapy for Neuroleptic Malignant Syndrome: A case report

**DOI:** 10.1192/j.eurpsy.2023.2213

**Published:** 2023-07-19

**Authors:** A. Osca Oliver, M. V. López Rodrigo, V. Ros Fons, M. Palomo Monge, A. Osca Oliver, M. F. Tascón

**Affiliations:** 1 Unidad de Psiquiatría; 2 Hospital Nuestra Señora del Prado, Talavera de la Reina, Spain

## Abstract

**Introduction:**

Neuroleptic malignant syndrome (NMS) is a rare syndrome observed in around 0.2% of psychiatric patients. This syndrome consists of the presentation of muscular rigidity, tachycardia, hyperpyrexia, leukocytosis and elevated levels of CPK. Any antipsychotic drug, including atypical ones, can cause this syndrome. This being an idiosyncratic response to dopamine receptor antagonist medications.

The use of ECT in patients suffering from NMS is very effective, seeing the progressive resolution of the picture in the first sessions.

**Objectives:**

The patient was a 43-year-old man, whose somatic history only highlights hypothyroidism, and according to his psychiatric history, he was diagnosed of mental retardation and paranoid schizophrenia. He was a resident of a group home. And he was recently admitted to a mid-stay psychiatric unit. During this admission, the responsible doctor added haloperidol to his medication regimen. His other medications at the time were; valproic acid, risperidone and trazodone. A few days later, the patient began to present a dysthermic sensation (presenting a temperature of 39ºC) and drowsiness. A laboratory tests and a chest X-ray was performed, highlighting: leukocytosis and an increase in elevated creatine phosphokinase (CPK).

Due to his recent exposure to 2 different antipsychotics, with fever, rigidity, and elevated CPK, we considered NMS. Antipsychotics were withdrawn and supportive measures were started. Within the next 2 days, his CPK level began to decline and the fever and leukocytosis resolved. But without resolving muscle rigidity. At the same time, he began to exhibit staring, negativism and prejudice delusions. Therefore, electroconvulsive therapy sessions were started.

**Methods:**

.

**Results:**

After the third session, his catatonic symptoms increased to better; he became more verbal, with less negativism and the psychotic symptomatology ceased. After the fifth session of ECT, he returned to his initial level and was able to walk to the bathroom without assistance and perform other activities of daily living.

**Conclusions:**

It is extremely important that professionals specialized in psychiatry become familiar with ECT and consider this technique as treatment in cases of neuroleptic malignant syndrome.

**Disclosure of Interest:**

None Declared

